# Supplementary material for: The Evolution and Characterization of the RNA Interference Pathways in Lophotrochozoa
Source: Genome Biol Evol. 2024 May 7;16(5):evae098. doi: 10.1093/gbe/evae098 (PMC11114477; doi:10.1093/gbe/evae098)
Supplement: evae098_Supplementary_Data [file evae098_supplementary_data.zip › Supplementary_materials.zip!/selected_miRNAs/NoGe_selected_miRNAs/mirdeep_pdf_files/NMRB01000470.1_Notospermus_geniculatus_strain_Ushimado_scaffold470,_whole_genome_shotgun_sequence_14117.pdf]

Provisional ID	:	NMRB01000470.1_Notospermus_geniculatus_strain_Ushimado_scaffold470,_whole_genome_shotgun_sequence_14117
Score total	:	258
Score for star read(s)	:	3.9
Score for read counts	:	249.9
Score for mfe	:	1.1
Score for randfold	:	0
Score for cons. seed	:	3
Total read count	:	502
Mature read count	:	487
Loop read count	:	0
Star read count	:	15

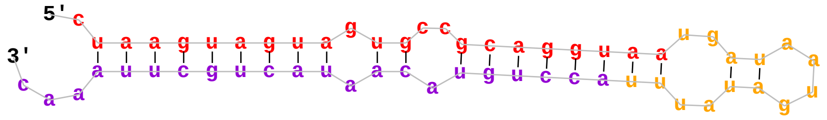

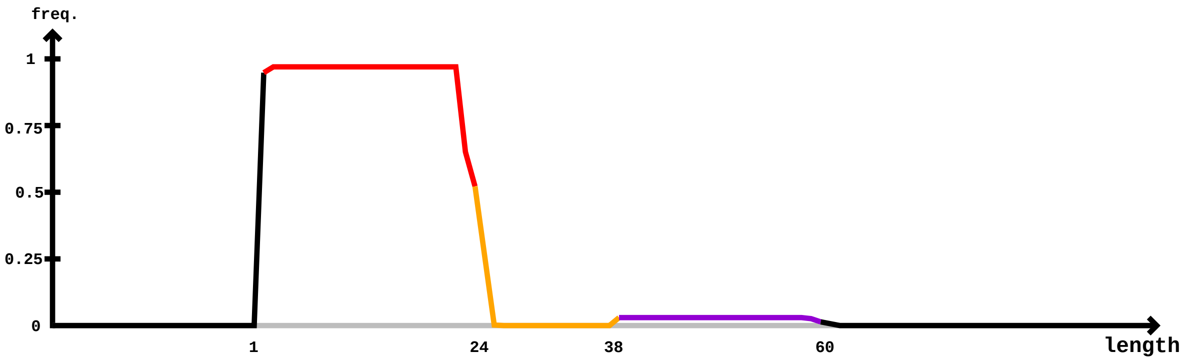


```
                    Mature                  Star
5'- auuuggcguuauugucucgacuaaguaguagugccgcagguaaugauaaugauauuuaccuguacaauacugcuuaaacgagcugaugaccauaucgaccugcccacgucaag  -3'   obs
    auuuggcguuauugucucgacuaaguaguagugccgcagguaaugauaaugauauuuaccuguacaauacugcuuaaacgagcugaugaccauaucgaccugcccacgucaag         exp
    ...(((((((..(.((((..(((((((((.((..((((((((..((....))..)))))))).)).)))))))))..))))))))))).)))....(((........)))...   reads   mm   sample
    ....................cuaaguaCuagugccgcaggu........................................................................   14      1    Mm1
    ....................cuaaguGguagugccgcaggua.......................................................................   1       1    Mm1
    ....................cuaaguaguagugccgcaggua.......................................................................   1       0    Mm1
    ....................cuaaguaguagugccgcagguaa......................................................................   7       0    Mm1
    .....................uaaguaguagugccgcagguaU......................................................................   1       1    Mm1

    ....................cuaaguaCuagugccgcaggu........................................................................   13      1    Mm2
    ....................cuaaguaguagugccgcaggu........................................................................   13      0    Mm2
    ....................cuGaguaguagugccgcaggu........................................................................   1       1    Mm2
    ....................cuaaguaguagugccgcaggua.......................................................................   5       0    Mm2
    ....................cuaaguaguagugccgcagguU.......................................................................   2       1    Mm2
    ....................cuaaguaguagugccgcaggCa.......................................................................   1       1    Mm2
    ....................cuaaguaguagugccgcagguaU......................................................................   7       1    Mm2
    ....................cuaaguaguagugccgcagguaa......................................................................   19      0    Mm2
    ....................cuaaguaguagugccgcagguaUu.....................................................................   1       1    Mm2
    ....................cuaaguaguagugccgAagguaau.....................................................................   1       1    Mm2
    ....................cuaaguaguagugccgcGgguaau.....................................................................   1       1    Mm2
    ....................cuaaguaguagugccgcagguaau.....................................................................   6       0    Mm2
    ....................cuaaguaguagugccgcagguaauU....................................................................   1       1    Mm2

    ....................cuaaguaguagugccgcaggG........................................................................   1       1    Fm2
    ....................cuaaguaguagugccgcaggu........................................................................   22      0    Fm2
    ....................cuaaguaguagugccgcagguaa......................................................................   15      0    Fm2
    ....................cuaaguaguagugccgcagguaU......................................................................   13      1    Fm2
    ....................cuaaguaguagugccgcagguaUu.....................................................................   19      1    Fm2
    .....................uaaguaguagugccgcagguU.......................................................................   10      1    Fm2

    ....................cuaaguaguagugccgcaggu........................................................................   31      0    Fm1
    ....................cuaaguaguagugccgGaggu........................................................................   1       1    Fm1
    ....................cuaaguaCuagugccgcaggu........................................................................   11      1    Fm1
    ....................cuaaguaguagugccgcCggu........................................................................   1       1    Fm1
    ....................cuaaguaguagugccgcaggua.......................................................................   36      0    Fm1
    ....................cuaaguaguaAugccgcaggua.......................................................................   1       1    Fm1
```

```
                    Mature                              Star
auuuggcguuauugucucgacuaaguaguagugccgcagguaaugauaaugauauuuaccuguacaauacugcuuaaacgagcugaugaccauaucgaccugcccacgucaag
....................cuaaguaguaCugccgcaggua.......................................................................   1   1   Fm1
....................cuaaguaguagugcUgcagguaa......................................................................   6   1   Fm1
....................cuaaguaguagugccgcagAuaa......................................................................   1   1   Fm1
....................cuaaguaguagugccgcagguaU......................................................................  13   1   Fm1
....................cuaaguaguagugccgcagguaa......................................................................  62   0   Fm1
....................cuaaguaguagugccgcagguaUu.....................................................................  15   1   Fm1
....................cuaaguaguagugccgcGgguaau.....................................................................   1   1   Fm1
....................cuaaguaguagugccgcagguaau.....................................................................  19   0   Fm1

....................cuaaguaCuagugccgcaggu........................................................................  13   1   Mm3
....................cuaaguaguagugccgcaggu........................................................................  12   0   Mm3
....................cuaaguaguagugccgcaggua.......................................................................   3   0   Mm3
....................cuaaguaguagugccgcagguU.......................................................................   1   1   Mm3
....................cuaaguaguagugccgcagguaU......................................................................   2   1   Mm3
....................cuaaguaguagugccgcagguaa......................................................................  10   0   Mm3
....................cuaaguaguagugccgcagguaau.....................................................................   3   0   Mm3
....................cuaaguaguagugccgcagguaUu.....................................................................   2   1   Mm3
....................cuaaguaguagugccgcagguaaA.....................................................................   2   1   Mm3

....................cuaagGaguagugccgcaggu........................................................................   1   1   Fm3
....................cuaaguaguagugccgcaggu........................................................................  16   0   Fm3
....................cuaaguaCuagugccgcaggu........................................................................  10   1   Fm3
....................cuaaguaguagugccgcaggua.......................................................................   3   0   Fm3
....................cuaaguaCuagugccgcagguaa......................................................................   2   1   Fm3
....................cuaaguaguagugccgcagguaa......................................................................  25   0   Fm3
....................cuaaguaguagugccgcagguaau.....................................................................   5   0   Fm3
....................cuaaguaguagugccgcagguaaA.....................................................................   2   1   Fm3
....................cuaaguaguagugccgcagguaUu.....................................................................   1   1   Fm3
.........................................................accuguacaauacugcuuaa....................................   2   0   Fm3
.........................................................accuguacaauacugcuuaaa...................................   6   0   Fm3
.........................................................accuguacaauacugcuuaaac..................................   7   0   Fm3
```
